# Chitosan-Based (Nano)Materials for Novel Biomedical Applications

**DOI:** 10.3390/molecules24101960

**Published:** 2019-05-21

**Authors:** Gregor Kravanja, Mateja Primožič, Željko Knez, Maja Leitgeb

**Affiliations:** 1 Laboratory for Separation Processes and Product Design, Faculty of Chemistry and Chemical Engineering, University of Maribor, Smetanova ul. 17, 2000 Maribor, Slovenia; gregor.kravanja@um.si (G.K.); mateja.primozic@um.si (M.P.); zeljko.knez@um.si (Ž.K.); 2 Faculty of Medicine, University of Maribor, Taborska ulica 8, 2000 Maribor, Slovenia

**Keywords:** chitosan, antimicrobial, nanomaterials, mode of action, brain drug delivery carrier, biomedicine

## Abstract

Chitosan-based nanomaterials have attracted significant attention in the biomedical field because of their unique biodegradable, biocompatible, non-toxic, and antimicrobial nature. Multiple perspectives of the proposed antibacterial effect and mode of action of chitosan-based nanomaterials are reviewed. Chitosan is presented as an ideal biomaterial for antimicrobial wound dressings that can either be fabricated alone in its native form or upgraded and incorporated with antibiotics, metallic antimicrobial particles, natural compounds and extracts in order to increase the antimicrobial effect. Since chitosan and its derivatives can enhance drug permeability across the blood-brain barrier, they can be also used as effective brain drug delivery carriers. Some of the recent chitosan formulations for brain uptake of various drugs are presented. The use of chitosan and its derivatives in other biomedical applications is also briefly discussed.

## 1. Introduction

**Figure 1:**
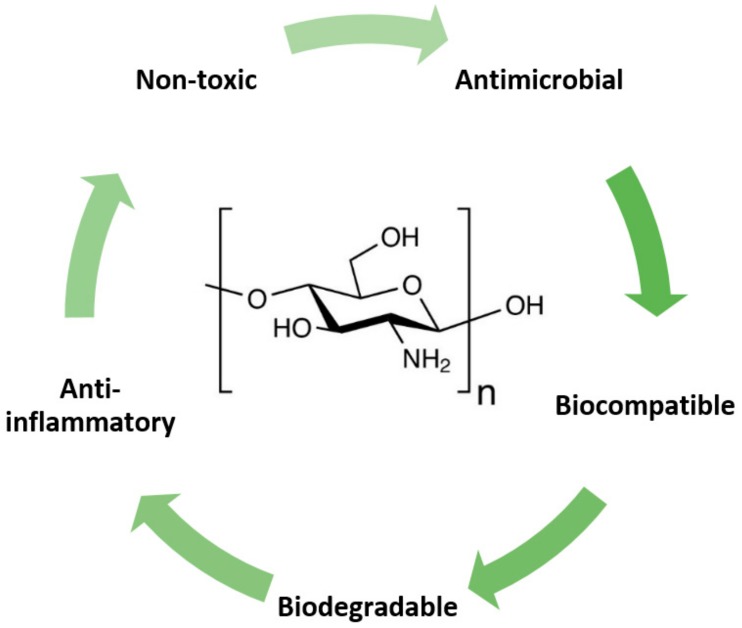
Unique biological properties allow the use of chitosan in many biomedical applications.

Chitosan is a naturally-occurring, linear polysaccharide produced from chitin by deacetylation in the solid state under alkaline conditions, or by enzymatic hydrolysis of chitin deacetylase. It is considered to be the second largest renewable biomaterial after cellulose in terms of utilization and distribution [1]. In recent years, chitosan and its derivative biomaterials have attracted significant attention in the biomedical field, owing to their unique biological properties. Some of the most noted properties of chitosan in the medical context are its non-toxicity, biodegradability, biocompatibility, and immunoenhancing, antitumoral, antibacterial and antimicrobial activity (Figure 1). The biodegradability of chitosan was proven both *in vitro* and *in vivo*, where macromolecules were split into several smaller sections of monomers [2]. Chitosan and its enzymatic degradation products could safely interact with living cells without any adverse effect in the body. Chitosan could decrease cholesterol absorption [3], interrupt the chain oxidation process by sifting free radicals [4], and act as an antimicrobial and antibacterial against many bacteria, yeasts and fungi [5]. It is suggested that the antimicrobial activity of chitosan and its derivative biomaterials relies on numerous factors like the degree of deacetylation, molar weight, pH, the presence of metal cations, pKa and microorganism species [6]. Chitosan-based nanoparticles can also inhibit tumor cell growth by inducing apoptosis, with a high permeability and retention effect. Particles up to about 100–200 nm in size can be adopted by receptor-mediated endocytosis, while larger particles have to be taken by phagocytosis [7]. Chitosan, either alone or mixed with other polymers, active agents and metallic nanocomposites, has been extensively used in many biomedical applications, including in wound dressing as an antimicrobial agent [8], in drug delivery as a nano-sized carrier to target tumor tissue while minimally affecting sites of normal tissue, [9], for gene delivery [10], hemodialysis [11] and dentistry [12] as well as for absorbable structures for immobilized enzymes [13]. 

In this article, we present several emerging biomedical applications including chitosan-based biomaterials, where two of most commonly investigated by the scientific community are highlighted: antimicrobial wound dressing and drug delivery including formulations for brain uptake. Firstly, insights about the antibacterial effects and mode of action of chitosan-based nanomaterials and their role as carriers for drug delivery are presented. Chitosan is proposed as an excellent antimicrobial wound dressing nanomaterial, either in its native form or formulated with antimicrobial substances to additionally increase its antimicrobial effect. Since chitosan and its derivatives can enhance drug permeability across the blood-brain barrier, they can also be used as excellent brain drug delivery carriers. Some of the recent chitosan formulations for brain uptake of various drugs are presented. The use of chitosan and its derivatives in other biomedical applications is also briefly discussed.

## 2. Antimicrobial Nature of Chitosan

### 2.1. Mode of Action

**Figure 2:**
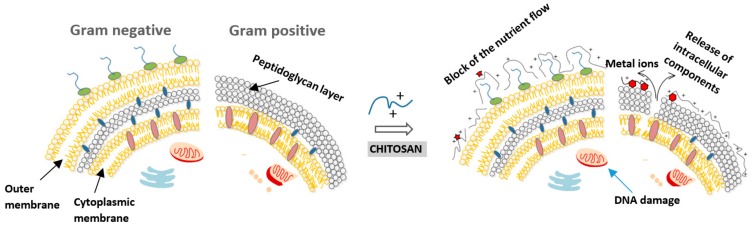
Four proposed models for the action of chitosan on Gram-positive and -negative bacteria: The polycationic nature of chitosan causes the release of intercellular components, binding to bacterial DNA (inhibition of mRNA), blocking the nutrient flow and chelation of essential metals.

Although the exact antimicrobial mechanism of chitosan and its derivatives is not yet fully understood, several hypotheses about the mode of action have recently been proposed and accepted (Table 1 and Figure 2). The most generally accepted hypothesis is that positively-charged amine groups (NH^3+^) of glucosamine interact with the negatively charged surface of bacteria, causing leakage of intracellular constituents that results in cell death [14]. Other possibilities include the binding of chitosan with DNA that inhibits mRNA after it penetrates to the nuclei of microorganisms. A study of *Echerichia coli* using a confocal laser microscope suggests that chitooligomers were present within the cell and probably prevent DNA transcription [6]. Another option is a chelating effect by chitosan that binds essential metals and thereby inhibits microbial growth. It is well known that chitosan has excellent metal-binding abilities, where amino charged groups interact with metals [15]. The interaction between amino groups and divalent ions like Ca^2+^ and Mg^2+^ present in the microorganism cell wall prevents the production of toxins and inhibits bacterial growth. The fourth hypothesis presents chitosan as a blocking agent that blocks nutrients and oxygen from entering the cell.

### 2.2. Effect of Factors on the Antimicrobial Activity of Chitosan

Several factors are recognized to affect the antimicrobial activity of chitosan and can be divided into three main categories: environmental factors, fundamental factors of chitosan and factors affecting different types of microorganism.

#### 2.2.1. Environmental Factors

Environmental pH is one of the most important factors affecting the antimicrobial activity of chitosan and its derivatives. Chitosan shows its antimicrobial effect only in an acidic medium, since it possesses poor solubility at high pH (pH ≥ 6.5). The reason for that may lie in the fact that the majority of amino groups become uncharged near pH 7 [24]. To overcome this limitation, chitosan has been systematically modified to yield derivatives with improved aqueous solubility. It has three types of reactive functional groups that can be further modified with quaternary ammoniumyl, guaindinyl, carboxyalkyl, long alkyl chains and thiol groups [25]. Another environmental factor includes the effect of the temperature during storage of chitosan. To date, the effect of temperature has been evaluated only for chitosan but not its derivatives. One study reveals that a chitosan solution stored for 15 weeks at 4 °C showed the highest antimicrobial effect against *Listeria monocotgenes*, *Salmonella enterica*, *Staphylococcus aureus* and *E. coli*, compared to one stored at 25 °C [26].

#### 2.2.2. Fundamental Factors

In the case of lower molecular weight, the size and conformation of chitosan appear to be fundamental, since mobility, attraction and ionic interaction are easier with small chains than with larger ones, resulting in effective binding to the membrane surface [5]. However, the relation between antibacterial activity and molecular weight also depends on the type of microorganism. One study showed that the growth of gram-positive and gram-negative bacteria was inhibited only by a molecular weight lower than 470 kDa, while toward *E. coli*, the opposite effect was found [27]. In the same way, but differing in intensity, the chitosan antimicrobial effect was improved as the degree of acetylation decreased [5]. In the process of deacetylation, acetyl groups from chitin are removed to form amino groups and determine the degree of deacetylation (the content of free amino groups in chitosan) [28]. In a study by Takahashi et al. [27] using mannitol salt agar and conductometric assay, the influence of the degree of deacetylation was investigated against the inhibition of *S. aureus*. A lower degree of deacetylation more successfully inhibited the growth of bacteria. In contrast, Byun et al. reported that the activity of chitosan, prepared from the ground shell with a degree of deacetylation of 81.6% is greater than that of chitosan prepared from the entire shell with a degree of deacetylation of 62% [29]. The reason for that could be that chitosan with a higher degree of deacetylation has more positively-charged free amino groups influencing the antimicrobial characteristics.

#### 2.1.3. Type of Microorganism

It was found that chitosan exhibits diverse inhibitory efficiency against different bacteria and fungi. The mode of antibacterial activity is a very complex process that varies between Gram-positive and Gram-negative bacteria, based on differences in cell structure (Figure 2). Gram-negative bacteria have an outer membrane that contains lipopolysaccharides that provide bacteria with hydrophilic surface properties. The outer membrane serves as a barrier against macromolecules and hydrophobic toxins. In the case of Gram-positive bacteria, the surface is made of peptidoglycans and teichoic acid, crucial for many membrane-bound enzymes to function. Many studies, with some exceptions, suggest that a higher inhibitory effect can be found in Gram-negative bacteria [30]. Younes et al. showed that the bactericidal effect was further enhanced for Gram-negative bacteria when chitosan molar weight decreased [31]. As with the bacterial effect, chitosan activity is assumed to be fungistatic rather than fungicidal [5]. There is no need for chemical modification of chitosan to activate its antifungal activity. The reason for the antifungal activity of chitosan is its polycationic nature. The formation of chelates with trace elements makes them unavailable for normal growth of fungi. Next, negatively charged membrane phospholipids interact with positively-charged chitosan, which increases the permeability of the membrane. The result is leakage of cellular content and subsequently, cell death. Chitosan may also penetrate the cell wall of fungi and bind to its DNA. However, chitosan nanoparticles increase its antifungal effect significantly [32]. Chitosan nanoparticles prepared from various concentrations of low molecular weight and high molecular weight were also found to provide better inhibitory activity against *Candida albicans* and *Fusarium solani* compared to chitosan in the solution form [33]. In the search for new agents with antifungal activity against *Candida*, chitosan obtained from *Pleurotus* spp. showed promising results. With regard to chitosan biofilms, important for biomedical applications, it was found that reduced biomass and metabolic activity in both the adhesion phase and in the mature biofilms display no significant difference for the strains of *Candida parapsilosis sensu stricto* and *Candida tropicalis* [33], using different molecular weights of chitosan [30]. 

Influence of environmental, fundamental factors of chitosan and factors affecting different types of microorganism are presented in Table 2.

In many studies, the assay conditions for determination of chitosan minimal inhibitory concentration (MIC) have not been standardized; it is therefore difficult to make effective comparisons between studies. Table 3 presents a set of several uniform and standard measurements, taking into account the effect of acetylation degree, molecular weight, and pH on MIC.

## 3. Chitosan in Biomedical Applications 

Chitosan-based nanomaterials have attracted significant attention in a wide variety of biomedical fields because of their unique chemical properties, including desired biodegradability, compatibility and non-toxicity. In tissue engineering, chitosan is a suitable biomaterial to construct extracellular tissue matrixes [45]. It can be used as a carrier for brain drug delivery and for various ocular therapeutic molecules such as drugs, genes and proteins in ophthalmology that deal with visual system diseases. Another medical application involves its use in renal failure and hemodialysis [46]. Chitosan is widely used as a carrier in delivering active agents and drugs [47], in gene and cancer therapy [48], in biosensor monitoring and bio imaging [49,50]. In dentistry, chitosan acts as an anti-plaque agent and can interfere with all microorganisms, while exhibiting antibacterial activity [51]. As an antimicrobial and antifungal agent, chitosan is most commonly used in wound dressing, since it possesses excellent tissue-adhesive properties [52]. 

### 3.1. Wound Dressing 

The healing process of a wound comprises a cascade synchronized events: hemostasis, inflammation, migration, proliferation and remodeling [53]. To ensure an effective wound healing process, many labs around the word started to develop antimicrobial wound dressings [54]. Among the many synthetic or natural materials in use, chitosan is an excellent choice on account of its distinctive properties, since it possesses intrinsic antimicrobial properties and offers the capacity to deliver extrinsic antimicrobial agents to the infected area. Chitosan can be easily fabricated into the desired asymmetric, porous skin scaffolds [55], hydrogels [56], sponges [57], membranes [58] and films without toxic chemicals [59]. Hydrogels are characterized by their high capacity to store water within their structure and can be used to moisten the infected area [60]. The swelling properties of hydrogels in saline solution need to be characterized in detail for potential biomedical applications. Their drawbacks include poor mechanical properties and the need for additional upgrading with secondary dressing [61]. Sponges are nothing more than foams with high open porosity and swelling properties, providing excellent matrixes to most wounded areas. However, these may incite skin maceration and are inappropriate for treatment of third-degree wounds [62]. Films used in biomedical applications must be resistant to bacteria and allow therapeutic monetization [63]. Nevertheless, this type of dressing could adhere to the wound bed and exudate accumulation [64]. Membranes are a commonly used type of wound dressing, as they can provide three-dimensional matrices with high surface-volume ratio to guarantee a good nutrient supply and cell proliferation. Chitosan-based antimicrobial wound dressing can be fabricated either alone, by using only native molecules, or upgraded and incorporated with antibiotics (ciprofloxacin, gentamicin, sulfadiazine or tetracycline), metallic antimicrobial particles (nAg, nCu, nZnO and nTiO_2_) and natural compounds and extracts (*Juglena regia*, *Salix alba* leaves, honey, *Aloe vera* etc.) ^®^ [54,55,56,57,58,59].

In the native form, many commercial wound dressings are available on the market, including HidroKi^®^, Chitpack^®^, Patch^®^, Tegasorb^®^ and KytoCel^®^ [54]. In order to increase the antimicrobial effect, chitosan is often functionalized with antibiotics. Antibiotics incorporated into chitosan matrices can interfere with the function of the bacterial structure or with metabolic pathways. They ca also interfere with cell wall biosynthesis [65], block key metabolic pathways as they mimic folic acid structure [66], or interfere with protein synthesis and can inhibit replication or transcription processes [67]. Recently, metallic antimicrobial particles, incorporated into chitosan scaffolds/bandages, have received much interest in clinical scenarios ranging from nAg based wound dressing, to nAg coated medical devices [68]. The main problems associated with metallic nanoparticles are their genotoxic, oxidative and cytotoxic effects [69]. However, the toxic effect can be significantly reduced by using chitosan-based biomaterials as carriers or surface coatings [70]. Among the metallic nanoparticles, nAg have attracted the most attention, owing to their broad inhibitory activity against many antibiotic-resistant bacteria [71]. Anishia et al. [72] presented antimicrobial sponges composed by chitosan, hyaluronic acid and nAg as wound dressing to treat diabetic foot ulcers infected with drug-resistant bacteria. Antibacterial activity of the prepared sponges was analyzed using *E. coli*, *S. aureus*, methicillin-resistant *S. aureus* (MRSA), *P. aeruginosa* and *Klebsiella pneumoniae*. Sponges containing higher nAg (0.005%, 0.01% and 0.02%) concentrations showed antibacterial activity against drug-resistant bacteria. In a recent survey, metallic composites were united with natural products to increase their antibacterial nature and biocompatibility [73]. An increasing number of chitosan-based wound dressings have been formulated with natural compounds and extracts in order to increase antimicrobial activity [74,75]. *A. vera* is a natural compound that has been extensively studied in combination with chitosan for antimicrobial dressing applications [76]. Silva et al. [77] highlighted the use of chitosan and *A. vera* membranes as active wound dressing materials useful for skin repair. Nguyen et al. [78] prepared composite sponges that were made with 10% curcumin and from chitosan and gelatin with various ratios. An *in vivo* study indicated better wound closure in wounds treated with curcumin-composite sponge than those with composite sponge without curcumin and an untreated group. Honey is one of the natural products that displays high bactericidal activity [79]. A range of honey-impregnated wound dressings is already on the market, including MediHoney [80], Actilite [81] and Algivon [82]. Honey from the Manuka tree is the most promising type of honey, since it contains a non-peroxide component and can sustain bacterial growth inhibition over time [83]. To date, there has been no publication about chitosan-based wound dressings containing Manuka tree honey. Table 4 presents several of the proposed and used wound dressings, prepared either as native chitosan or modified and formulated with other antimicrobial substances to additionally increase its antimicrobial effect. 

### 3.2. Tissue Engineering for Bone Regeneration

Three-dimensional porous scaffolds for bone tissue engineering must be biocompatible and allow osteogenesis. A variety of techniques have been developed to mimic the microstructure and properties of natural mineralized materials. Still, facile and rapid fabrication of large-size bulk materials with high calcium content under ambient conditions remains a major challenge. During gelation, a controllable inorganic gradient distribution forms, along with mineralization, where urea was used, and spontaneously hierarchically ordered hydrogel microstructures were formed. This fabrication route takes only hours to complete the gelation and mineralization processes [104]. 

Composite scaffolds have been fabricated using a coaxial electrospinning technique to prepare gelatin-chitosan core-shell structured nanofibers. An arginine-glycine-aspartic acid (RGD)-like structure was formed to mimic the organic component of the extracellular matrix of natural bone. Later, by a wet chemical method, hydroxyapatite was deposited on the surface of the prepared-shell structured nanofibers. Hydroxyapatite is the major mineral constituent of natural bone. Gelatin-chitosan core-shell structured nanofibers improved the mineralization efficiency of hydroxyapatite compared to chitosan nanofibers [105]. The design of biologically-active scaffolds is focused on the application of cell-adhesive proteins or bioceramic nanoparticles to produce a cell-sensitive surface. Therefore, trace metals found in the living organisms were used to prepare biocompatible chitosan hydrogels. These were modified by copper (II) ions through complexation interactions and yielded a less stable cytocompatible to more stable cytotoxic structure for a copper-chitosan system [106,107]. 

From the perspective of antibacterial effects, Zhou et al. [108] synthesized a biodegradable rhBMP-2-loaded zein-based scaffold with SBA-15 and hydroxypropyltrimethyl ammonium chloride chitosan (HACC) nanoparticles incorporated into the scaffolds. Results showed that the sample zein-HACC-S20 exhibited long-lasting antibacterial activity against *E. coli* and *S. aureus*. In another study, scaffolds were made of poly-ε-caprolactone (50 kDa), containing chitosan and vancomycin as an antimicrobial agent for bone regeneration purposes. The scaffolds sustained vancomycin release for more than 2 weeks. The scaffolds were tested against Gram-positive (*S. aureus*) and Gram-negative (*E. coli*) bacteria after 24 h of incubation with full growth inhibition for *S. aureus* [109]. Although in this subsection only limited insight is provided on using chitosan for bone tissue engineering, there is immense future clinical potential in fabricating composite materials using chitosan as an antimicrobial and mechanical support biomaterial for better cell proliferation.

### 3.3. Chitosan as a Brain Drug Delivery Carrier 

**Figure 3:**
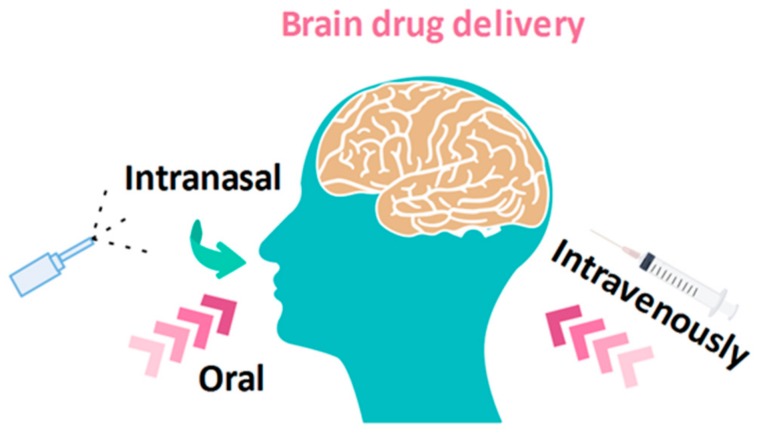
The demand for more effective therapeutics has led to the development of multiple strategies to reach the brain tissue.

Peptide and protein drug delivery systems could be improved by chitosan and its derivatives [110]. The molecular dynamics simulation results revealed that the native conformation of insulin was stabilized by the chitosan polymers [111]. Chitosan has also been extensively studied in brain drug delivery [112]. The reason is that it can enhance drug permeability across the blood-brain barrier by affecting the tight junction. Chitosan nanoparticles can be absorbed on the negatively charged cell membrane, owing to the positive charge on the surface and can increase the residence time on the nasal mucosa. The delivery of drugs from the nasal cavity to the brain can thus be improved. Chitosan nanoparticles may be surface modified with tumor-targeting peptides like chlorotoxin and transferrin, which can further improve the capacity for targeting a brain tumor [113]. The demand for more effective therapeutics has led to the development of multiple strategies to reach the brain tissue (Figure 3). Some of these rely on the transient disruption of the blood-brain barrier by using osmotic pressure, ultrasound or pharmacological entities, while others are based on intra-cerebro-ventricular (ICV) infusion, convection-enhanced delivery, intra-cerebral injection or the use of implants [114].

The first nasal formulations with a chitosan absorption enhancer to induce the brain’s uptake of neuroactive agents were proposed in 2008 [112]. Nasal formulations with the presence of chitosan or its derivatives were prepared as anti-ischemic drugs, anti-Alzheimer’s drugs, anxiolytic drugs, anti-Parkinson’s drugs, antimigraine drugs, anti-neurodegenerative drugs, antibiotic drugs, anti-nociceptive drugs, antiepileptic drugs and anti-HIV drugs. They have many advantages over liquid preparations: e.g., they are easy to handle, can be used as carriers for protein or other unstable drugs and provide a lengthy residence time of the drug in the nasal cavity. Chitosan may be used in its unmodified form or in the form of chitosan salts or chitosan derivatives. Different chitosan formulations using different preparation techniques and drugs were studied for the treatment of the same neuropathological conditions. Some of them are mentioned here. 

The N,N,N-trimethyl chitosan (TMC) is a positively charged, quaternized hydrophilic derivative of chitosan. It has excellent solubility over the entire pH range compared to chitosan and is completely biodegradable and biocompatible, while possessing greater bioadhesivity than chitosan [115]. It also improves the nasal residence time of the nanocarrier formulation and helps to enhance permeability across the nasal mucosal membrane [116]. It has been used for formulations in different forms, such as neuronanoemulsions (NNEs) or hydrogels. 

Ropinirole-dextran sulfate (ROPI-DS) nanocomplex-loaded, flaxseed oil-based NNEs and TMC-modified mNNEs have been developed for direct nose-to-brain drug delivery, with the objective of providing controlled drug release for the treatment of Parkinson’s disease (PD). Direct nose-to-brain drug delivery in a mouse model has achieved significantly high drug concentrations in the brain compartment [117]. Furthermore, thermosensitive hydrogel from pluronic F127 (PF127) and TMC was developed as a drug delivery system for the anticancer drug docetaxel (DTX) to glioblastoma multiforme (GBM). With intratumoral drug delivery to the tumor, it is very unlikely to achieve uniform distribution and it has a low retention time. Retention time can be increased by using a hydrogel delivery system for encapsulation of the drug [118]. The addition of TMC to the gel system increased the porosity of the gel network as observed by SEM, and that led to higher release of DTX from the gel. Cytotoxicity data revealed that the hydrogel system can effectively kill cancer cells, in contrast to free DTX [119]. 

For treatment of PD, pramipexole dihydrochloride-loaded chitosan nanoparticles (P-CNs) were developed in another study for effective brain targeting via the non-invasive nasal route. Nanoparticles were prepared using chitosan and sodium tripolyphosphate by the ionic gelation method. P-CNs significantly enhanced antioxidant status in the form of increased superoxide dismutase and catalase activity, along with increased dopamine levels in the brain [120]. Ropinirole hydrochloride (R-HCl) is a non-ergoline dopamine (D2) receptor agonist used in PD therapy. The conventional oral tablet dosage forms of R-HCl available on the market exhibit low oral bioavailability, due to its extensive first-pass metabolism in the stomach after oral administration. A study was done to increase the brain uptake of R-HCl through chitosan nanoparticles prepared by a conventional emulsification crosslinking method. Coupling nanotechnology with surfactant coating on the chitosan nanoparticles resulted in enhanced brain targeting of the encapsulated drug [121]. 

The most common neurodegenerative disorder to cause dementia is Alzheimer’s disease (AD). Type-2 diabetes (T2D) is one of the major risk factors associated with AD. Similarities have been found in the molecular mechanisms that underlie the respective degenerative development in the two diseases. Saxagliptin (SAX) is a dipeptidyl peptidase-4 enzyme inhibitor molecule explored for its activity in AD therapy. The dipeptidyl peptidase-4 enzyme increases the level of glucagon-like peptide-1 (GLP-1) and ameliorates T2D [122]. Because of its extreme hydrophilicity, SAX is unable to permeate the blood-brain barrier by conventional therapy modalities. Chitosan-L-valine (CSV)-based nanoparticles loaded with SAX were developed, utilizing the large amino acid transporter (LAT-1) at the blood-brain barrier, which helps in the transport of amino acids and amino acid-like molecules into the brain cells. *In vivo* application of the formulation resulted in enhanced drug delivery in the brain compared to the suspension of SAX, which showed no detectable amount even after 24 h [123]. 

A biodegradable chitosan-poly(lactide-co-glycolide) (PLGA) drug was synthesized in the form of a nanoformulation as a therapeutic approach for the delivery of neuroprotective drugs to the brain in the treatment of epilepsy [124], which has been defined as a recurrent and unpredictable set of chronic neurological disorders in the normal brain [125]. L-pGlu-(1-benzyl)–L-His–LProNH2 (NP-355) and L-pGlu-(2-propyl)–L-His–L-ProNH2 (NP-647)-loaded PLGA nanoparticles were prepared and surface modified with chitosan to provide mucoadhesive properties for successful intranasal delivery of drugs to the brain. The *in vivo* studies showed that recovery was significantly improved after intranasal administration of NP-355 and NP-647 NPs, as compared to un-encapsulated NP-355 and NP-647 [124]. Also, carboxymethyl chitosan nanoparticles as a carrier to deliver carbamazepine (CBZ) (CBZ-NPs) was developed for intranasal administration [126]. CBZ is used in the clinical treatment of seizure disorders, trigeminal neuralgia and, most recently, manic depression. The *in vivo* results showed that both encapsulation of CBZ in nanoparticles and the nasal route resulted in enhanced drug bioavailability and brain targeting characteristics [127]. 

Another possibility is to produce lipid microparticles (LMs), coated with chitosan and containing the polyphenol resveratrol. Specifically, for central nervous system diseases, resveratrol has been reported to be effective against neurologic disorders such as AD and PD, brain ischemia and epilepsy. *In vivo* studies were performed on rats. Such an LMs system attained complete targeting of resveratrol to the CNS via a direct nose-to-CSF route, resveratrol being undetectable in the bloodstream of rats after nasal administration of the chitosan-coated resveratrol-loaded LMs [128]. Recent studies of chitosan formulations for brain uptake of different drugs are presented in Table 5.

### 3.4. Other Biomedical Applications 

Chitosan formulations as drug delivery carriers are not limited to delivering active agents to the brain, but can be used for many other delivery purposes in biomedicine. As mentioned before, the low solubility of chitosan in biological systems limits its use as a drug delivery carrier [110]. Therefore, derivation of chitosan to improve its hydrophilicity is a promising path. A glycol-chitosan-based carrier is suitable and most popular for drug delivery in biological systems, owing to its water solubility and biocompatibility [143,144]. On the other hand, chitosan is also insoluble in most organic solvents, and is therefore inappropriate as a carrier for hydrophobic drugs [145]. To overcome this deficiency of chitosan, various derivation methods were developed to increase the encapsulation efficiency of hydrophobic components. When release of a drug cannot be performed by using simple drug dissolution processes such as diffusion, in anionic drug delivery systems, anionic polymeric excipients (e.g., chitosan) can be used. The mucoadhesive nature of chitosan and its ability to temporarily open tight epithelial junctions allow its application in drug delivery systems for different epithelia [146,147]. Mucosal surfaces are the most common and convenient routes for delivering drugs to the body. However, macromolecular drugs, such as peptides and proteins, are unable to overcome the mucosal barriers, since they are usually degraded before reaching the bloodstream. To overcome this problem, the use of nanostructures based on the mucoadhesive polysaccharide chitosan is a promising alternative [148]. Chitosan-based nanoparticles have several advantages that place them among the potential carriers for genes and other nucleic acid delivery systems. Because of the positive charge of chitosan, interaction with negatively charged nucleic acid molecules forms a polyelectrolyte complex [149], which protects nucleic acids from degradation caused by nuclease [150]. Various methods have been reported for the preparation of chitosan-based protein-loaded particles. The most commonly used preparation method is emulsification with cross-linking, where the use of organic solvents and crosslinkers can negatively affect protein activity [148]. 

To improve the protein-sponging ability of chitosan, a variety of methods has been used—e.g., incorporation of different moieties (imidazole [149] histidine [151] etc.). Chitosan has been used to transfect a range of different cell types *in vitro* and *in vivo*. *In vitro,* the most commonly used cell lines that have been transfected using chitosan-pDNA complexes are HEK293, A549, HeLa and COS-1 [152]. Additionally, chitosan was successfully used as a carrier of RNAi in chitosan-RNAi complexes for transfection of CHO-K1, HEK293, H1299 and HepG2 cells [152]. Gene therapeutics can be delivered in a number of ways for clinical translation [153], but innovative methods involved the incorporation of complexes into a scaffold, with the aim of spreading them to the defective site [154,155].

Chitosan and chitosan nanocomposites have also been used in bioimaging, biomedical devices, sensors, etc. Darder at al. [156] reported that chitosan–montmorillonite nanocomposites constitute excellent materials for the development of bulk-modified potentiometric sensors for anionic detection in aqueous samples. A graphene/AuNPs/chitosan electrode showed high electrocatalytic activity toward H_2_O_2_ and O_2_ and could be used for the construction of a glucose biosensor [157]. A GOx- *O*-(2-hydroxyl)propyl-3-trimethylammonium chitosan chloride NP-conjugated anion membrane biosensor showed good sensitivity, reproducibility, reusability, low cost and an easy-to-operate system for quantification of glucose in the system [158]. Shakya and Nandakuma [159] prepared the Au-GOx-based glucose sensor, which showed good stability for up to 30 days, with high sensitivity (8.91 µA mM^−1^ cm^−2^) and fast response times. Palladium nanoparticles modified with chitosan oligosaccharide (COS) were functionalized with RGD peptide, which improves particle accumulation in MDA-MB-231 breast cancer cells and results in enhanced photothermal therapeutic effects under an 808-nm laser. The RGD peptide-linked, COS-coated palladium nanoparticles (Pd@COS-RGD) destroyed the tumor effectively under 808-nm laser illumination at a power density of 2 W cm^−2^. They act as an ideal nanotheranostic agent for enhanced imaging of and therapy for tumors using a non-invasive near-infrared laser [160]. Karagozlu et al. [161] reported that QMW-chitosan oligomers and WMQ-chitosan oligomers (Q, - glutamine M -methionine and W - tryptophan) exerted a protective action on C8166 cells against the cytolytic effects of an HIV-1RF strain. Additionally, the saquinavir-loaded chitosan nanoparticles indicated the potency of the system as an effective anti-HIV system, since both strains of HIV – NL4-3 and Indie-C1 were found to respond to the saquinavir -chitosan delivery system [162]. Many of these emerging biochemical applications, including chitosan-based nanomaterials, are presented in Table 6.

## 4. Conclusions and Future Trends

Chitosan is one of the most abundant polysaccharide polymers, and is capable of significant antimicrobial activity against a wide variety of fungi and bacteria. Despite many proposed hypotheses and contributing effects, there is no consensus on the mechanism of action for its antimicrobial activity. This could be explained by the fact that, in most studies, the assay conditions have not been standardized and it is thus difficult to make a good comparison between them. The most widely accepted hypothesis is that positively-charged amine groups (NH_3_^+^) of glucosamine interact with the negatively charged surface of bacteria, causing leakage of intracellular constituents that results in cell death. 

In the present review, chitosan has been presented as an ideal renewable agent for the fabrication of antimicrobial wound dressings, either alone in its native form or upgraded and incorporated with antibiotics, metallic antimicrobial particles, natural compounds and extracts. The incorporation of metallic nanoparticles into chitosan-based matrices has been reported as a novel idea to increase and sustain antimicrobial effect. Prior to clinical application *in vivo*, the toxicity of nanoparticles still needs to be solved. There has been no effort made to fully evaluate its efficiency in an infectious area *in vivo* and *in vitro*. Similarly, there are many opportunities for future studies of chitosan use in combination with natural compounds and extracts. Further improvement to chitosan-based wound dressings are also needed. These include development of new smart dressings containing sensors and therapeutic molecules that are released at the same time as the microbial growth. In this concept, a wound dressing system releases bioactive compounds when any change in the medium occurs, including pH, temperature or UV due to initial bacterial activity.

Brain drug delivery is another biomedical application where chitosan and its derivatives as nano-biodegradable carriers play an important role. A wide variety of chitosan-based nanoparticles has proven to improve the therapeutic efficacy in various brain diseases, owing to its fine biological properties, modifiability and effective uptake by intranasal mucosal cells to tumor cells. There are still challenges to solve regarding the toxicity of some chitosan derivatives, which could serve as carriers of material for nose-to-brain drug delivery. Moreover, in the case of chitosan and protein complexes, the interaction between polysaccharides and proteins, the phase behavior and colloidal properties of complexes between chitosan, a positively-charged polysaccharide, and proteins still need to be investigated. In acidic pH, chitosan is highly protonated, which stabilizes the complexes. Furthermore, the addition of NaCl leads to a reduction in both size and zeta-potential and facilitates complex aggregation. 

In conclusion, chitosan-based nanomaterials are often present in today’s clinical applications and are expected to have an even greater impact on emerging biomedical applications.

## Figures and Tables

**Table 1:** Recent studies of various hypotheses on the mechanism of chitosan antimicrobial action.

Antimicrobial Mechanism	Findings
Polycationic nature of chitosan	The interaction between positively-charged chitosan molecules and negatively charged microbial cell membranes leads to leakage of intracellular constituents [16,17] The binding neutralizes and reverses the surface charge of bacteria Cationic groups increase *E. coli* membrane permeability and membrane lysis [18]
Binding to bacterial DNA (inhibition of mRNA)	Binding to bacterial DNA leads to inhibition of mRNA and consequently protein synthesis The low molecular weight (≤50 kDa) chitosan and nano-sized particles can penetrate the bacteria cell wall and inhibit DNA transcription [14] Binding of chitosan to bacterial DNA was commonly investigated for gene delivery [19] The mechanism of DNA binding ability and its antimicrobial activity are not yet fully understood
Chelation agent (nutrients and essential metals)	Chitosan selectively binds essential metals and thereby inhibits microbial growth and the production of toxins [20] Higher inhibitory efficiency at high pH where positive ions are bonded to chitosan Activates defense processes in host tissue [17] Acts as a water-binding agent that inhibits several enzymes [21]
Blocking agent	Chitosan can form a layer on the surface of the bacteria cell and prevent nutrients from entering the cell [22] Blocking the oxygen path and inhibiting the growth of aerobic-type bacteria [23]

**Table 2:** Influence of three main factors affecting the antimicrobial activity of chitosan.

Factors Influencing Antimicrobial Activity	Findings
**Environmental Factors**	
pH	Higher antimicrobial activity at low pH (ionized amino groups) [34] At pH ≤ 6, positively-charged amino groups interconnected with proteins, fatty acids, phospholipids and consequently with negative charged bacterial membrane [35]
Temperature	Different temperatures influence chitosan antimicrobial activity during storage [36] Temperature affects chitosan viscosity and molecular weight [36]
**Fundamental Factors**	
Molecular weight	High molecular weight chitosan could stack on the bacterial surface and block nutrient transport, resulting in cell death [37] Lower molecular weight (≤50 kDa) chitosan could penetrate the membrane surface and bind with DNA, thus inhibiting synthesis of mRNA [38]
Degree of acetylation	A higher positively-charged chitosan is associated with the degree of acetylation [39] A 30–40% degree of acetylation produced the highest antibacterial activity against *S. aureus* and *E. coli* [40]
**Type of Microorganism**	
Gram-positive bacteria	Comprises peptidoglycan and teichoic acid responsible for structural constancy of cell wall [41]
Gram-negative bacteria	It is suggested that chitosan possesses the strongest bactericidal effect on Gram-negative bacteria [41] Gram-negative bacteria have a cell wall of a thick peptidoglycan layer that has a highly negative charge [40]
Fungi	Antifungal activity decreased with increasing molar weight and decreasing degree of acetylation [31] Chitosan in solution or chitosan films displayed varying efficiency on fungal growth [42]

**Table 3:** Minimal inhibitory concentration (MIC) of chitosan against three types of microorganisms.

Type of Microorganism	pH	Mw (kDa)	Degree of Acetylation (%)	MIC (µg/mL)	Ref.
**Gram-positive**					
*Bacillus cereus*	5.5	43	6	60	[22]
*Bacillus cereus*	6	2.3–224	16–48	80–2000	[43]
*Bacillus megaterium*	5.9	28–1670	-	500–800	[26]
*Lactobacillus brevis*	5.9	224–1106	-	500–1000	[26]
*Lactobacillus bulgaricus*	5.9	28–1670	-	up to 1000	[26]
*Listeria monocytogenes*	6	49–1100	2–53	150	[44]
*Staphylococcus aureus*	5.9	28–1106	-	800–10000	[26]
**Gram-negative**					
*Escherichia coli*	6	49–1100	-	100–500	[44]
*Escherichia coli*	5.9	28–1670	2–53	800–1000	[26]
*Escherichia coli*	6	3–224	16–48	30–2000	[43]
*Enterobacter aerogenes*	5.5	43	6	60	[22]
*Pseudomonas aeruginosa*	6	49–1100	2–53	150–200	[44]
*Pseudomonas fluorescens*	5.5	43	6	80	[22]
*Salmonella typhimurium*	6	49–1670	2–53	1500–2000	[44]
*Vibrio cholera*	6	49–1100	2–53	200	[44]
**Fungi**					
*Botrytis cinerea*	-	-	-	10	[6]
*Candida lambica*	5.5	43	6	400	[22]
*Drechstera sorokiana*	-	-	-	10	[6]
*Fusarium oxysporum*	6	49–1100	2–57	500–2000	[44]
*Microsporum canis*	-	-	-	1000	[6]
*Micronectriella nivalis*	-	-	-	10	[6]
*Trichophyton equinum*	-	-	-	2500	[6]

**Table 4:** Recent chitosan-based wound dressings prepared either as native chitosan or modified and formulated with other antimicrobial substances.

	Type	Findings	Tested Microorganisms	Ref.
**Native Chitosan-Based Biomaterials**				
Chitosan/PVA/starch	Membrane	Excellent cell growth and proliferation	*Escherichia coli,* *Staphylococcus aureus*	[84]
Chitosan/β-cyclodextrin polymer	Sponge	Controlled swelling and drug delivery	*Staphylococcus aureus,* *Escherichia coli*	[85]
Chitosan	Hydrogel	Superb antifungal and antimicrobial effects	*Pseudomonas aeruginosa,* *Escherichia coli,* *Fusarium solani*	[86]
Chitosan	Membrane	Epithelialization rate was increased	*Pseudomonas aeruginosa,* *Staphylococcus aureus*	[58]
Chitosan/PVP/nano-cellulose	Film	*In vitro* wound dressing application was significant	*Staphylococcus aureus*	[87]
Chitosan-distamycin and vancomycin	Films	80% degrees of deacetylation were optimal for eluting antibiotics	*Staphylococcus aureus*	[88]
**Chitosan and Antibiotics**				
Chitosan nanofiber mesh-gentamicin-loaded liposomes	Membrane	Antibacterial activity	*Escherichia coli*, *Pseudomonas aeruginosa, Staphylococcus aureus*	[89]
Chitosan/poly(2-hydroxyethyl acrylate)-levofloxacin	Sponge	The prepared dressing shows a significant inhibition zone of bacteria strains	*Methicillin- susceptible Staphylococcus aureus,* *Methicillin- resistant Staphylococcus aureus*	[90]
Chitosan-vancomycin	Aerogel	Low-density, large surface area	*Staphylococcus aureus*	[91]
Chitosan/sulfadiazine	Sponge	Antibacterial activity	*Escherichia coli,* *Staphylococcus aureus,* *Bacillus subtilis*	[92]
**Chitosan-Entrapped Metallic Nanoparticles**				
Chitosan/sodium alginate-Cu	Hydrogel	Safe to use in contact with living cells	*Methicillin-resistant Staphylococcus aureus,* *Escherichia coli*	[93]
Quaternized chitosan-nAg	Film	Property with antibacterial effects	*Escherichia coli, Staphylococcus aureus, Pseudomonas aeruginosa, Candida albicans*	[94]
Chitosan-nAu	Film	nAu interacts with cell wall and inhibits mitochondrial membrane	*Staphylococcus aureus, Pseudomonas aeruginosa*	[95]
Chitosan/algetic acid-nZnO	Sponge	Potential to be an antibacterial topical hemostat	*Staphylococcus aureus*	[96]
Chitosan/gelatin- nFe_3_O_4_		Fe_3_O_4_ enhanced mechanical and antibacterial properties	*Escherichia coli,* *Staphylococcus aureus*	[97]
Chitosan/ECM-nTiO_2_	Composite	Faster regeneration of granulation tissue	*Escherichia coli, Staphylococcus aureus*	[98]
**Chitosan Entrapped with Plant Extracts**				
Chitosan- amorphophallus konjac plant	Film	Low cytotoxicity and inhibition of microbial penetration.	*Escherichia coli, Staphylococcus aureus,* *Pseudomonas aeruginosa*	[99]
Chitosan- *Hypericum perforatum*	Film	The highest strain value was obtained in 0.25% oil content films	*Escherichia coli,* *Staphylococcus aureus*	[100]
Chitosan- Aloe vera	Membrane	Promising wound dressing material	*Escherichia coli,* *Staphylococcus aureus*	[101]
Chitosan-thyme oil	Films	Antibacterial activity on all studied microorganisms	*Escherichia coli,* *Staphylococcus aureus,* *Klebsiella pneumoniae*	[102]
Poly(vinyl alcohol)/chitosan-honey	Hydrogel	Faster honey release rate at higher pH values	*Staphylococcus aureus*	[103]
Chitosan/gelatin-curcumin	Sponge	Enhances the formation of collagen and wound closure *in vivo*	*Pseudomonas aeruginosa*	[78]

**Table 5:** Recent studies of chitosan formulations for brain uptake of different drugs.

	Drug	Disease	Delivery	Ref.
Chitosan nanoparticles	Chlorotoxin and transferrin	Brain tumors	Intranasal	[113]
Chitosan nanoparticles	Pramipexole	Parkinson’s disease	Intranasal	[120]
**Flaxseed oil/N,N,N-trimethyl** chitosan neuronanoemulsion	Ropinirole-dextran sulfate	Parkinson’s disease	Intranasal	[117]
Pluronic F127/N,N,N-trimethyl chitosan hydrogel system	Docetaxel	Malignant glioma	Intracranial injection	[119]
Chitosan/L-valine -based nanoparticles	Saxagliptin	Alzheimer’s disease	Intraperitoneal route	[123]
Chitosan coated lipid microparticles	Resveratrol	Central nervous system diseases	Nasal administration	[128]
Poly-lactide-co-glycolide/chitosan nanoparticles	L-pGlu-(1-benzyl)–L-His–LProNH2 and L-pGlu-(2-propyl)–L-His–L-ProNH2	Epilepsy	Intranasal	[124]
Chitosan nanoparticles	Ropinirole hydrochloride	Parkinson’s disease	Intravenously via the dorsal tail vein	[121]
Carboxymethyl chitosan nanoparticles	Carbamazepine	Epilepsy	Intranasal	[127]
Methoxy poly(ethylene glycol)-grafted Carboxymethyl chitosan nanoparticles	Doxorubicin	Malignant glioma		[129]
Poly-ε-caprolactone nanocapsules coated with chitosan	Simvastatin	Brain tumors	Intranasal	[130]
Chitosan-based mucoadhesive microemulsions	Diazepam	Epilepsy	Intranasal	[131]
Chitosan nanoparticles	Rotigotine	Parkinson’s disease	Intranasal	[132]
Chitosan nanoparticles	Genistein	Neurodegenerative diseases	Intranasal	[133]
Chitosan lipid nanoparticles	Risperidone	Schizophrenia	Intranasal	[134]
Nano lipid Vit E mixed with melted Gelucire 44/ 14/chitosan hydrogel formulation	Temozolomide	Metastatic melanoma and glioma	Intranasal	[135]
Chitosan-coated liposome dry-powder formulations	Ghrelin	Cachexia	Intranasal	[136]
Chitosan nanoparticles	Cyclovirobuxine D	Cardiovascular disease	Intranasal	[137]
Chitosan glutamate coated niosomes	Pentamidine	Alzheimer’s disease	Intranasal	[138]
Glycol chitosan coated nanostructured lipid carrier	Asenapine maleate	Schizophrenia and bipolar disorders	Intranasal	[139]
Nasal chitosan microspheres	Hydroxypropyl--cyclodextrin	Alzheimer’s disease	Nasal route	[140]
Chitosan oligosaccharide	Chitosan oligosaccharide lactate	Depression		[141]
Chitosan-grafted HPbCD intranasal EFV nanoparticles	Efavirenz	Neuro-AIDS	Intranasal	[142]

**Table 6:** Selected emerging chitosan-based biomedical applications.

Matrix	Biomedical Application	Findings	Ref.
Zinc-chitosan nanoparticles	Treatment of acute lymphoblastic leukemia	Induced apoptosis in human acute T-lymphocyte leukemia through activation of tumor necrosis factor receptor CD95	[163]
Sodium alginate beads with olive oil and coating with chitosan	*Helicobacter pylori* infections	Controlled release of active Clarithromycin	[164]
Timolol maleate-loaded galactosylated chitosan nanoparticles	Ocular delivery of timolol maleate	*In vitro* transcorneal permeation study and confocal microscopy showed enhanced penetration, and retention in the cornea	[165]
Modified glycol chitosan nanoparticles encapsulated camptothecin	Cancer therapy	Stable and effective drug delivery system in cancer therapy	[166]
Insulin-loaded lecithin/chitosan nanoparticles	Drug delivery system to the deep lung	Improved oral bioavailability, time-dependent release, and therapeutic activity	[167]
Chitosan grafted poly(ethylene glycol) methacrylate	Posterior eye diseases	Particles were found suitable from the cytotoxicity and hemocompatibility points of view	[168]
Palladium nanoparticles chitosan oligosaccharide (COS) functionalized with RGD peptide	Breast cancer therapy by imaging	Matrix acts as an ideal nanotheranostic agent for enhanced imaging and tumor therapy, using a non-invasive near-infrared laser	[160]
Graphene/AuNPs/chitosan electrode	Construction of a glucose biosensor	High electrocatalytic activity toward H_2_O_2_ and O_2_	[157]
Chitosan-RNAi complexes	Gene therapy	Transfection of CHO-K1, HEK293, H1299, HepG2 cells	[152]
Chitosan–montmorillonite nanocomposites	Biomedical sensors	Bulk-modified potentiometric sensors for anionic detection in aqueous samples	[156]
Chitosan-Au particles	Biomedical sensors to detect DNA	Low cost of preparation	[169]
Saquinavir-loaded chitosan nanoparticles	Effective anti-HIV system	Strains of HIV – NL4-3 and Indie-C1 were found to respond to delivery system	[162]
Magnetic chitin nanofiber composite	Immobilization of therapeutic enzyme	Immobilized chymotrypsin could be easily separated and recycled from the reaction system by magnetic force	[170]
